# Supplementary material for: A fairness assessment of mobility-based COVID-19 case prediction models
Source: PLoS One. 2023 Oct 18;18(10):e0292090. doi: 10.1371/journal.pone.0292090 (PMC10584164; doi:10.1371/journal.pone.0292090)

**SUPPLEMENTAL MATERIALS**

**Manuscript title:**

A fairness assessment of mobility-based COVID-19 case prediction models

**Manuscript #:** PONE-D-23-04556

The choropleth maps of the US in Figure S1 show the distribution of the prediction error rates as well as the distribution of the socio-economic and demographic features. To be able to compare them, we have created pairs of plots where the left-hand side represents the error rate distribution and the right-hand side a specific socio-economic or demographic metric. Since errors are computed monthly, we have represented the average error across all months in the period under analysis. The value-ranges for the color-coded socio-economic and demographic features in the choropleths were selected based on best trend visualizations. In the plots below, we show the visualizations for the 1-day regression model (Model 1). A visual exploration confirms the quantitative results discussed in the paper i.e., that areas with a higher rurality (dark green), lower income rates (white), lower education rates (white), older population rates (dark orange), lower population rates (white) and lower smartphone ownership rates (white), tend to be associated to higher error rates (darker red).

**Fig S1.** Spatial comparison of error rate and demographic features for Model 1. **a.** Error rate versus rural-urban. **b.** Error rate versus income. **c.** Error rate versus age. **d.** Error rate versus education. **e.** Error rate versus population. **f.** Error rate versus smartphone ownership. (These plots have been generated with Plotly open-source graphing libraries using base maps from OpenStreetMap. OpenStreetMap is open data, licensed under the Open Data Commons Open Database License (ODbL) by the OpenStreetMap Foundation (OSMF).)

**a.**


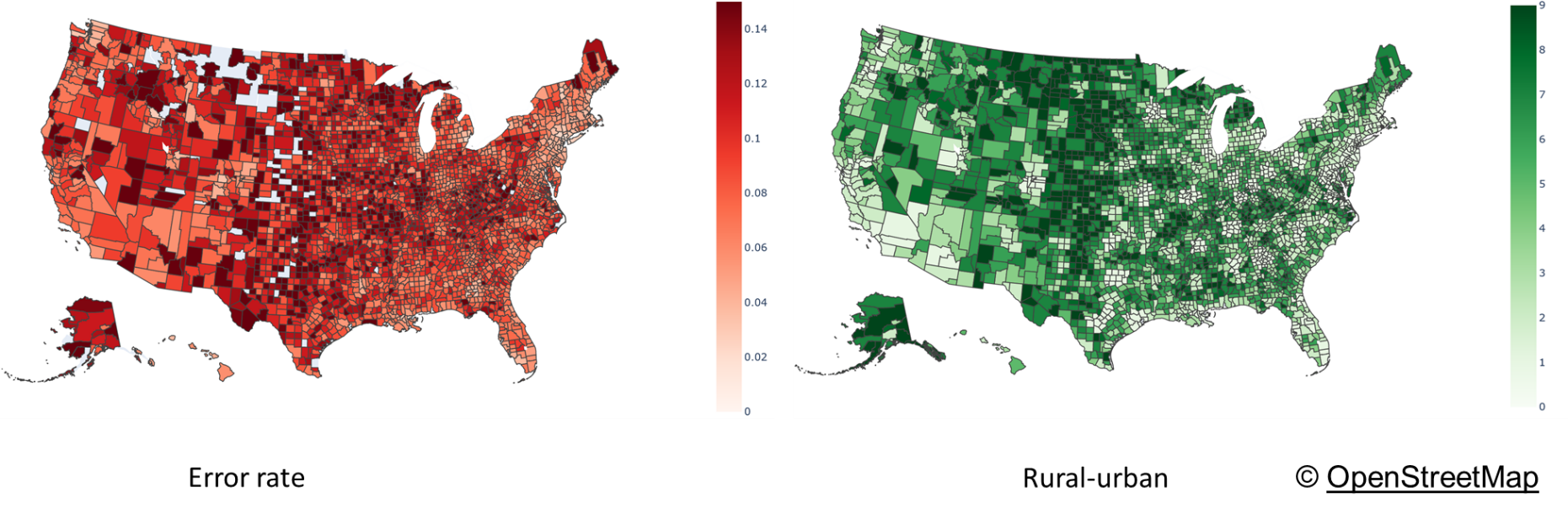


**b.**


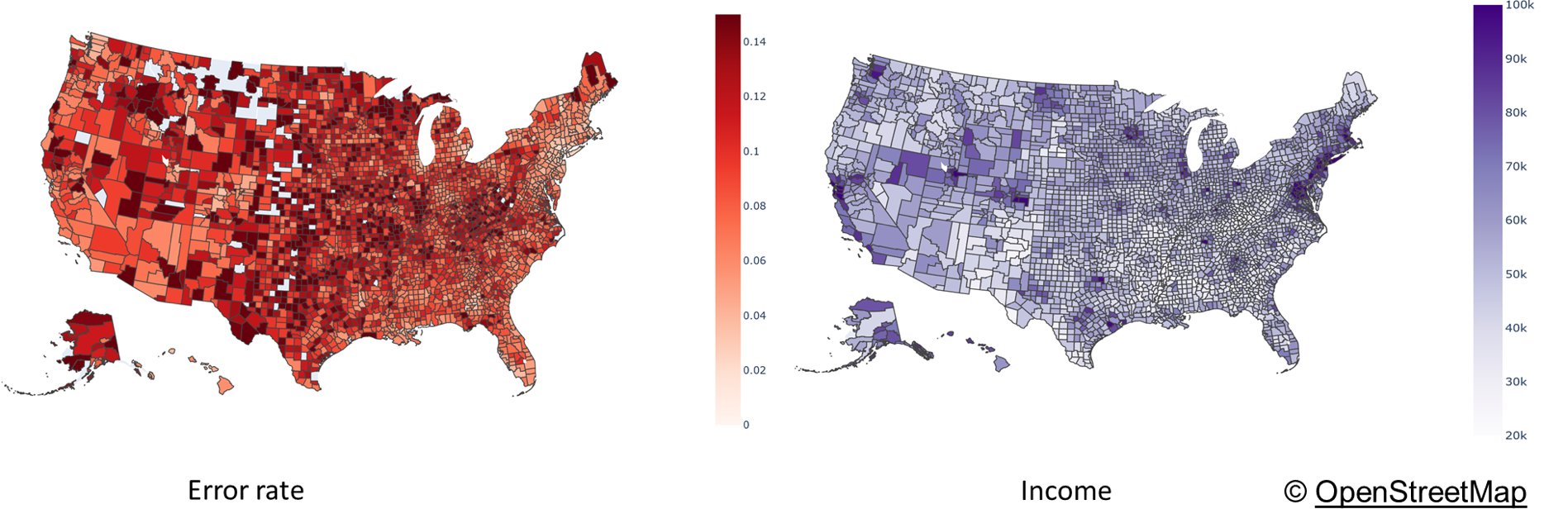


**c.**


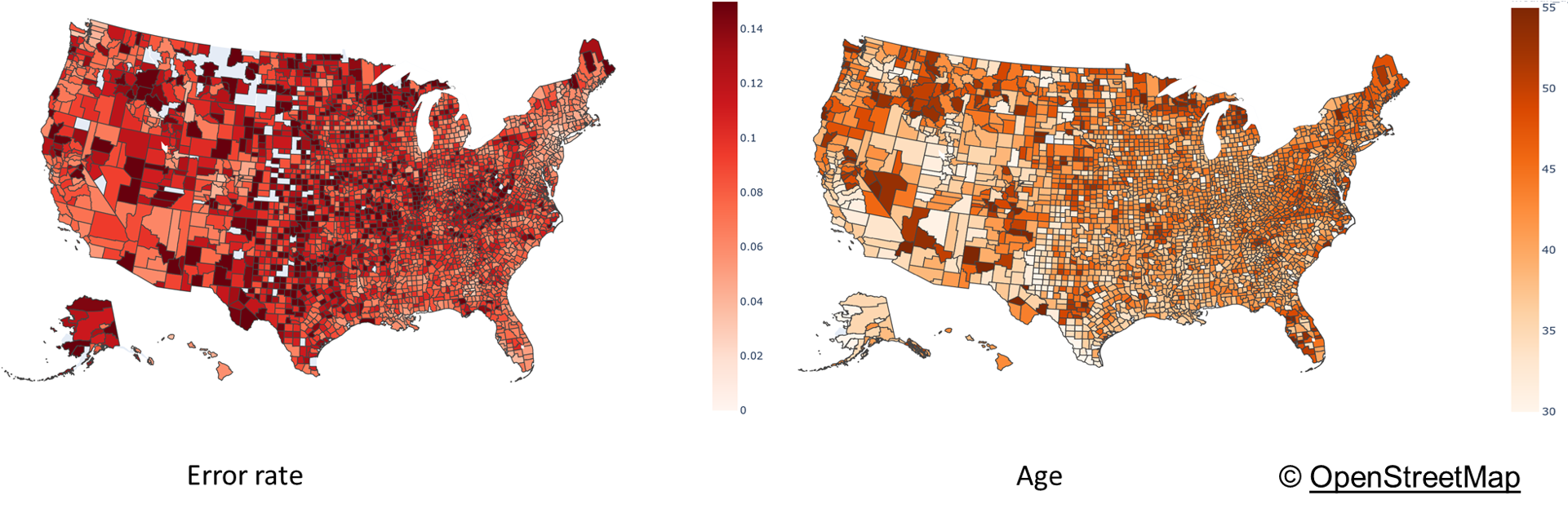


**d.**


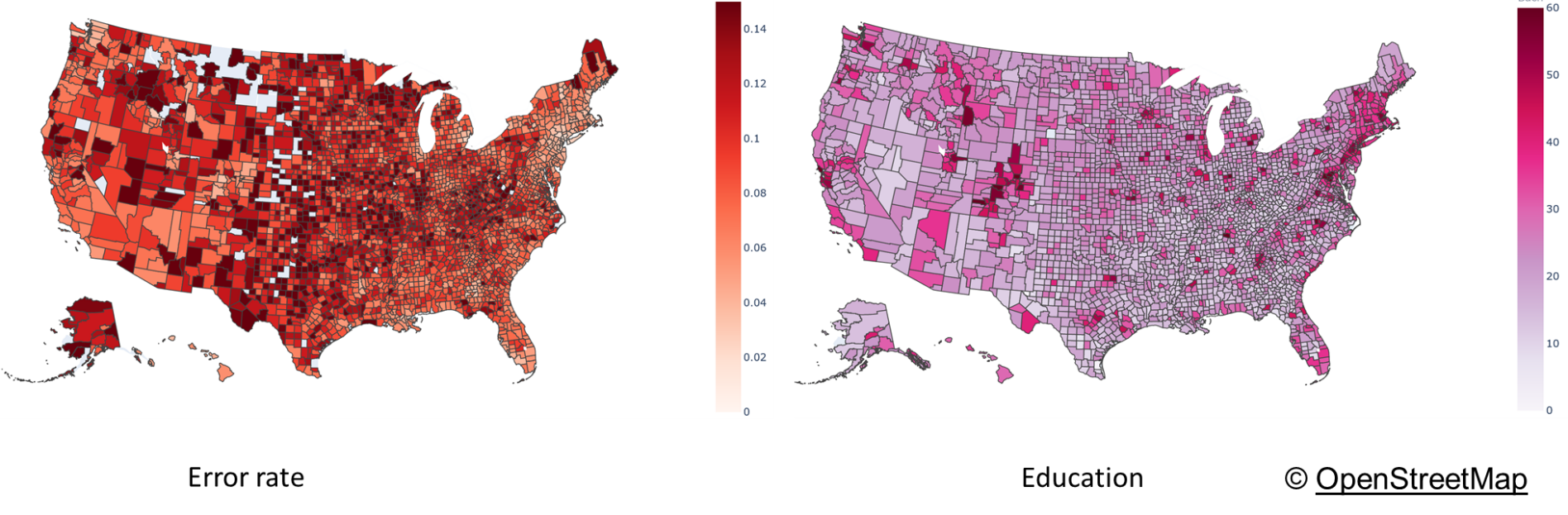


**e.**


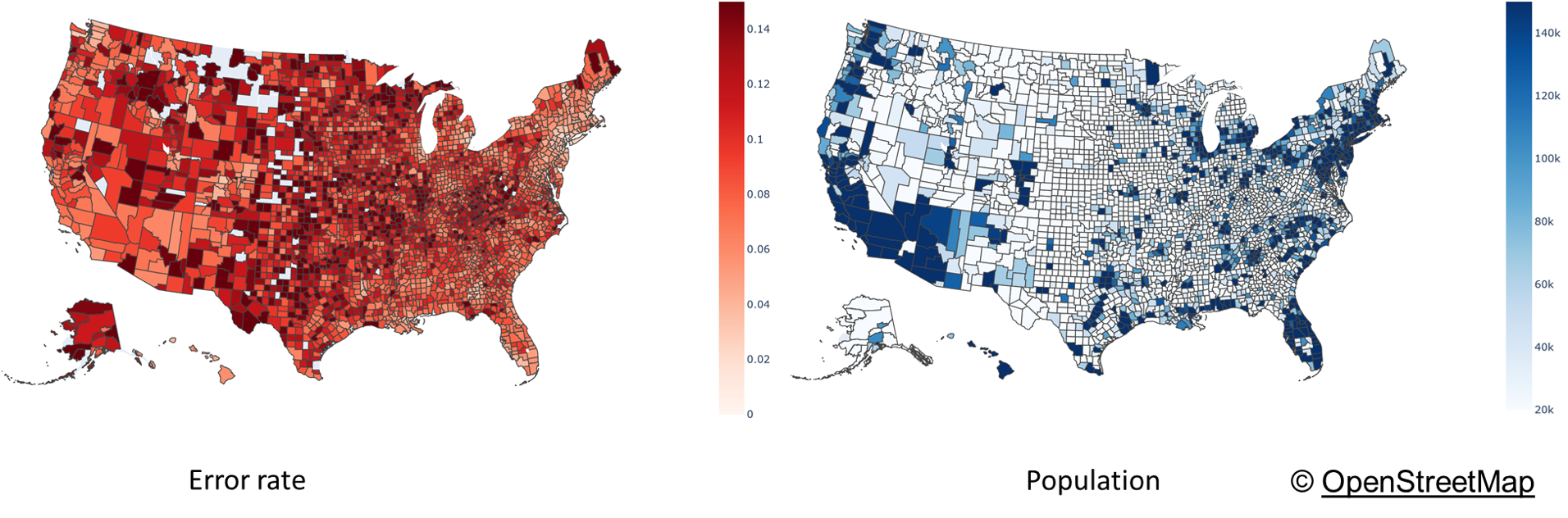


**f.**


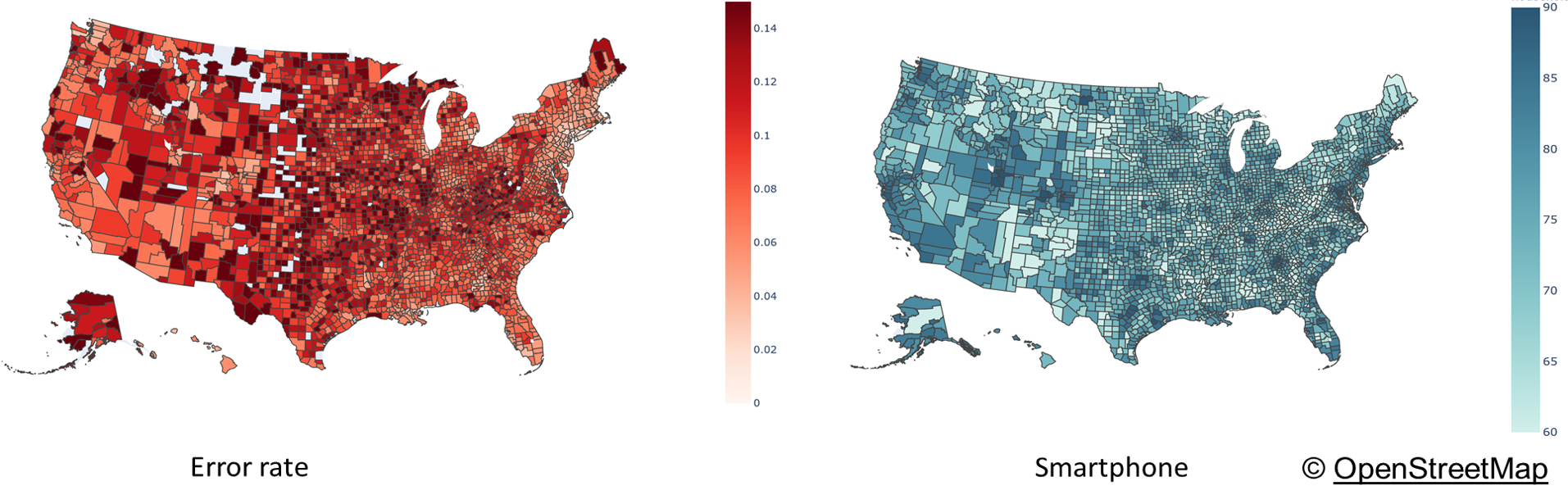

Supplement: S1 File — (DOCX) [file pone.0292090.s001.docx]
