# Supplementary material for: A fairness assessment of mobility-based COVID-19 case prediction models
Source: PLoS One. 2023 Oct 18;18(10):e0292090. doi: 10.1371/journal.pone.0292090 (PMC10584164; doi:10.1371/journal.pone.0292090)
Supplement: Attachment — Submitted filename: Authors responseR2-vfm8.23.docx [file pone.0292090.s003.docx]

**AUTHORS’ RESPONSE TO REVIEWER COMMENTS**

**Manuscript title:**

A fairness assessment of mobility-based COVID-19 case prediction models

**Manuscript #:** PONE-D-23-04556R1

The authors are grateful for the opportunity to resubmit the article. Please accept our thanks for the time and effort that the editors and reviewers spent reviewing our manuscript. All comments were seriously considered, and we conducted a thorough revision of the entire paper according to reviewers’ feedback. We hope the changes listed have made the manuscript suitable for publication and we look forward to your response.

Please note that due to the changes, line and page numbers referenced in the reviewers’ comments might be different compared to the updated version. Where applicable, page and line numbers are listed based on the new version which can be tracked.

**Editor:**

**1) Comment:** “Thank you for submitting your manuscript to PLOS ONE. After careful consideration, we feel that it has merit but does not fully meet PLOS ONE’s publication criteria as it currently stands. Therefore, we invite you to submit a revised version of the manuscript that addresses the points raised during the review process.

Both reviewers suggest that a minor revision of the manuscript may be sufficient for publication of the work. Please revise your manuscript according to the received recommendations, being careful to highlight the changes in the manuscript.”

- **Response:** We appreciate the time spent reviewing our paper by the department editor, and the reviewers. Our paper has been revised in response to your feedback. Please see below for our detailed responses to each particular comment.

**Reviewer 1:**

**2) Comment:** “Thank you to the authors for addressing these comments. I appreciate that the authors carefully addressed the limitations of their research. They also included choropleth maps and an interesting discussion on the error prediction rates from a spatial perspective. The authors also added an analysis of the difference between 1-day and 7-day predictions. I recommend a minor revision of the journal to adjust some formatting and grammar issues. Please address these minor comments:”

- **Response:** We appreciate your constructive comments. We hope the changes we have made have enhanced our paper to make it ready for publication.

**3) Comment:** “- 1. In Table 1, please include the appropriate unit of measurement either in the description or the individual cells. For example, Income could be” Median household income ($) “or “$70, 264.80”. I also observed that all the variables are at the county level, so this does not need to be repeated and should instead be in the Table description.

- **Response:** Thanks for your suggestions. We revised the table format accordingly.

**4) Comment:** “- 2. In Table 1, the description for smartphones is “percentage of the population at the county level with a bachelor degree and above.” Please correct this typo.”

- **Response:** Thank you for your comment. We have corrected the typo.

**5) Comment:** “- 3. In Figure 5, can you label which error map is Model 1 and Model 2? Also, I notice that there were more maps in the comment response that were not in the manuscript. Were these moved to supplemental information? If so, this should be referenced in the manuscript.”

- **Response:** Thank you very much for your comment. We have added the model number in the caption of Figure 5. In addition, we have created a supplemental materials file where we have added the remaining figures. As suggested by the reviewer, we have also added a reference to the supplemental materials in the text.

**6) Comment:** “- 4. I recommend that the authors review the grammar of the manuscript once more. There is an odd use of punctuation and run-on sentences which could be revised. I provided some examples but please review the whole manuscript.

E.g. “These results reveal that Model 2 – an ARIMAX with mobility data added as exogenous variable – performs better (i.e., has lower errors) in counties that share higher income, higher smartphone ownership, larger populations, and higher educational levels (see Figure 4 for weekly representations of the weekly correlations for some of these features).”

“Given that these findings are replicated across models 1 and 2, thus controlling for algorithmic bias, we posit that the model is unfair in part due to the bias in the mobility data used in the model, although bias in the way COVID-19 case data is collected, could also influence the outcome.”

**Response:** Thank you for your feedback. We have proofread the paper for grammatical and writing errors.

**Reviewer 2:**

**7) Comment:** “The authors addressed the majority of my comments; however, I still believe that they should better clarify how they interpret the correlation coefficients.

In particular, it is stated that “Correlation coefficient strengths can be interpreted differently across scientific fields, and authors should avoid overinterpreting associations”. As the main conclusion of the work is based on these correlation coefficients, it would be helpful to clarify how to interpret the obtained values in the context of their paper.

Since different interpretations of the correlation coefficient exist, the authors should better clarify the rationale of their choice and how they interpret those results. Specifically, they should better describe how the values in the table should be interpreted and avoid qualitative statements.”

- **Response:** Thank you for your comment. We mostly focused our fairness analysis and discussion on the significance and direction of the results i.e., p-values and positive or negative correlation signs. Following this approach, our results show statistically significant differences with all p-values under 0.05 that is, with at least 95% confidence in the results. We did not discuss strength in depth because as we state in the limitations section, coefficient strengths can be interpreted differently across scientific fields, and there is no formal agreement on how to define correlation strength for covid-19 studies. Nevertheless, we agree with the reviewer that our interpretation approach needs to be clearer to strengthen our analysis. Thus, we researched papers using spearman rank correlation in the contexts of medicine and big data analysis and agreed to select the coefficient of 0.3 as the threshold between high and low correlation (notation used in paper 2, see information below). To describe the strength of the correlation, we will use the words weak (below 0.3) and moderate (above 0.3) as defined in paper 1, see information below. We have also updated the results section accordingly to include a brief analysis of the correlation strength using the coefficient threshold selected based on existing work. We have also added language in the results section to clarify that the strength of the statistically significant relationship is low (weak). Finally, we have clarified our threshold selection in both the Methods and the Limitations sections. See edited text below.

In the Methods section:

“To discuss correlation strength, and based on prior work, we will use 0.3 as the correlation coefficient threshold between a high and low correlation **^51^**, or a weak and a moderate correlation **^52^**.”

In the Limitations Section:

“Based on prior work utilizing spearman rank correlation in the context of medicine and big data analysis, we have selected a correlation coefficient of 0.3 as the threshold between high and low correlation **^51^**, or weak and moderate correlation **^52^**.”

Paper 1: Akoglu, Haldun. "User's guide to correlation coefficients." Turkish journal of emergency medicine 18, no. 3 (2018): 91-93.

Paper 2: Xiao, Chengwei, Jiaqi Ye, Rui Máximo Esteves, and Chunming Rong. "Using Spearman's correlation coefficients for exploratory data analysis on big dataset." Concurrency and Computation: Practice and Experience 28, no. 14 (2016): 3866-3878.

**8) Comment:** “In addition, an extended description of the results should be added. Which sociodemographic features are more likely to cause bias, and which are almost negligible?”

**Response:** Thank you for your constructive comment. As mentioned in our prior response, we have focused the discussion of our results on both significance and direction of the coefficients i.e., p-values and positive or negative correlation signs. Given that the strength of the correlations found is weak, we posit that all socio-economic and demographic features are related to significant, albeit weak, bias. We do not observe statistically significant differences in the strengths across features. We do however see that correlation coefficients are smaller for 7-day predictions than 1-day predictions, which might point to more negligible bias for these models. Nevertheless, the statistical results do not support any feature being more biased than other. We have clarified this in the results discussion. See text below.

“Given that the strength of the correlations found is weak, we posit that all socio-economic and demographic features are related to significant, albeit weak, bias. We do not observe statistically significant differences in the strengths across features. We do however see that correlation coefficients are smaller for 7-day predictions than 1-day predictions, which might point to more negligible bias for these models. Nevertheless, the statistical results do not support any feature being more biased than other.”

**9) Comment:** “Why does the correlation vary so much among different months (for example, in Table 2 the Education in April is -0.25 and in December -0.06)?”

- **Response:**

We appreciate your feedback. As presented in related work (paper 1 and 2 below), earlier pandemic months showed strong, uniform mobility changes across the population, with large percentages of individuals reducing their mobility. We posit that these widespread changes make it easier to find associations (correlations) between mobility behaviors and other socio-economic and demographic data. However, during later pandemic months, and especially once vaccines were made available (from December onwards), mobility behaviors were more entropic, with population groups showing very diverse mobility behaviors across the US. As papers 1 and 2 show, these non-uniform changes made mobility data less useful in the analysis of covid-19 settings. We posit that these non-uniform changes make it harder for the analysis to find any associations or patterns of behavior between mobility and socio-economic and demographic data thus making the correlations much weaker. We have edited to results section to include this clarification. See text below.

“It is important to highlight that for certain socio-economic and demographic features in Tables 2 and 3, we observe some variance in the correlation coefficients across months, with earlier pandemic months showing higher coefficients. We posit that these might be due to mobility behaviors being more entropic later during the pandemic, which might make it harder to find associations. As prior work has shown, uniform mobility behaviors made mobility data more useful in predictive models at the onset of the pandemic than in later periods **^20,53^**.”

Paper 1: Badr, Hamada S., and Lauren M. Gardner. "Limitations of using mobile phone data to model COVID-19 transmission in the USA." The Lancet Infectious Diseases 21, no. 5 (2021): e113.

Paper 2: Gatalo, Oliver, Katie Tseng, Alisa Hamilton, Gary Lin, and Eili Klein. "Associations between phone mobility data and COVID-19 cases." The Lancet Infectious Diseases 21, no. 5 (2021): e111.

**10) Comment:** “Also, references to the tables (for example, line 248) and the content of Table 1 should be checked.”

- **Response:** Your feedback is greatly appreciated. We revised the typo in table referencing and in Table 1 content.
